# Influence of Maxillofacial Morphology on Temporomandibular Joint Degenerative Alterations and Condyle Position Assessed by CBCT in Class II Malocclusion Adult Patients—A Cross-Sectional Study

**DOI:** 10.3390/jcm14134499

**Published:** 2025-06-25

**Authors:** Sebastian Dygas, Izabela Szarmach, Ilona Radej, José Chaqués-Asensi

**Affiliations:** 1 Department of Orthodontics, Medical University of Bialystok, 15-274 Bialystok, Poland; izabela.szarmach@umb.edu.pl (I.S.); ilona.radej@umb.edu.pl (I.R.); 2 Independent Researcher, 41009 Seville, Spain; jchaques@jchaques.es

**Keywords:** temporomandibular joint disorders, jaw relation techniques, tomography, X-ray computed, orthodontics, computer-assisted technology, craniofacial abnormalities, malocclusion, angle class II

## Abstract

**Background/Objectives:** This cross-sectional analytical study investigated the relationship between the craniofacial morphology, condylar displacement, and degenerative changes in the temporomandibular joints (TMJs) in adult patients with class II skeletal malocclusion. To compare cephalometric variables, joint space dimensions, and centric slide measurements between patients with and without CBCT-confirmed TMJ degenerative alterations. **Methods:** Sixty adults with class II malocclusion were divided into two equal groups (n = 30) based on the presence or absence of TMJ degenerative changes on CBCT. Joint spaces were measured, condylar displacement was evaluated using a condylar position indicator (CPI), and cephalometric analysis was performed in both maximal intercuspation and centric relation. Statistical comparisons were performed using *t*-tests, chi-squared tests, and Pearson’s correlation analysis. Significance was set at *p* < 0.05. **Results:** Patients with degenerative TMJ changes exhibited significantly greater overjet (*p* = 0.0001) and a trend toward increased ANB angles (*p* = 0.055). The superior joint space was reduced on the right side (*p* = 0.031). Condylar displacements ≥ 2 mm were more frequent in the affected group and correlated with sagittal cephalometric discrepancies (45% vs. 24% in controls). **Conclusions:** Aggravated skeletal class II malocclusion with increased overjet could be associated with TMJ degenerative changes. CR-based cephalometry and CBCT evaluation may aid in diagnostic assessment, but longitudinal studies are needed to confirm the clinical relevance.

## 1. Introduction

The relationship between occlusion, the craniofacial morphology, and the positioning of the mandibular condyles within the temporomandibular joint (TMJ) has long been debated [1,2]. With an increasing number of adult patients presenting signs of TMJ disorder, this subject has gained substantial clinical relevance [3,4,5].

Inconsistencies in interpreting cephalometric parameters, particularly regarding their relationship with TMJ health and the condylar position, may lead to diagnostic discrepancies and influence treatment decisions. This is especially critical when managing patients with skeletal discrepancies or borderline treatment indications [6].

Centric relation (CR), a reproducible position of the condyles within the glenoid fossae, differs from maximal intercuspation (MIP), which represents habitual occlusion [1]. The difference between these positions, known as the centric slide (MIP-CR discrepancy), may influence the assessment of skeletal discrepancies if the magnitude of displacement exceeds clinically relevant thresholds [2,7,8].

Class II skeletal malocclusion, typically involving mandibular deficiency, has been linked to increased susceptibility to internal TMJ dysfunction and condylar remodeling. Altered loading patterns and joint compensation mechanisms in these patients may predispose them to early degenerative changes [3,6,9,10,11,12]. Recent reviews confirm the value of cone beam computed tomography (CBCT) in detecting degenerative changes and morphologic variability relevant to orthodontic patients [13,14].

The evaluation of the condylar position becomes particularly relevant in adults, who more frequently present with CBCT-detected degenerative joint disease (DJD) of the TMJ [8,15,16].

Despite increasing interest, no consensus has been reached regarding the relationship between the cephalometric morphology, centric slide, and degenerative TMJ changes in class II patients [13,17,18,19].

### Aim of the Study

This cross-sectional analytic study aimed to evaluate the relationship between the craniofacial morphology, centric slide, and the condylar position in adult class II malocclusion patients, comparing those with and without CBCT-confirmed TMJ degeneration.

We hypothesized that class II patients with TMJ degeneration would exhibit greater cephalometric discrepancies and increased condylar displacement due to centric slide compared to those without TMJ degeneration.

## 2. Materials and Methods

### 2.1. Study Population

The study was conducted in accordance with the Helsinki Declaration guidelines and received approval from the Institutional Bioethics Committee of the Medical University of Białystok, protocol code APK.002.37.2024 (approval date: 18 January 2024). All participants, or their legal guardians when relevant, completed all necessary informed consent documents prior to enrollment in the study.

The study group consisted of 60 patients with either full permanent dentition or interdental deficiencies due to the premature loss of permanent teeth. Gender was not considered in qualifying patients for the study. The inclusion and exclusion criteria are summarized in Table 1.

Patients were recruited consecutively and voluntarily from the university orthodontic clinic. Only individuals with TMJ sounds and mild-to-moderate TMJ pain were included so as to maintain sample homogeneity and avoid the confounding effects of severe DJD. Patients under 18 and those with unilateral condylar degeneration were excluded to ensure skeletal maturity and group symmetry.

A convenience sampling method was applied, enrolling all eligible patients who met the inclusion criteria during the recruitment period. No formal sample size calculation was performed due to the exploratory, cross-sectional design. However, the chosen sample size (n = 60) was based on previous CBCT-based studies on TMJ morphology in malocclusion patients and was considered sufficient to detect moderate effect sizes (Cohen’s d = 0.6), with power of 0.80 at α = 0.05, based on post hoc estimation [7,9,20,21]. Given the diagnostic specificity and single-center setting, larger-scale recruitment was not feasible.

The information obtained from the clinical examination was analyzed. Based on cephalometric measurements and CBCT imaging of the temporomandibular joints, patients were divided into 2 groups of 30 each—a control group (normal temporomandibular joints with no bone changes) and a study group with both joints affected and with degenerative alterations.

### 2.2. Imaging Studies

CBCT imaging was performed in patients with persistent TMJ symptoms and as part of treatment planning for skeletal discrepancies. Imaging was justified by clinical indications and aligned with the ALADAIP principles. Full CBCT acquisition parameters (scanner type, FOV, voxel size, etc.) and details regarding the patient’s position are provided in Appendix A.

Temporomandibular joint evaluation was conducted on 3D CBCT images, involving measurement of the anterior, superior, and posterior joint spaces, as well as an assessment of the articular surfaces to identify any signs of degenerative alterations.

**Figure 1:**
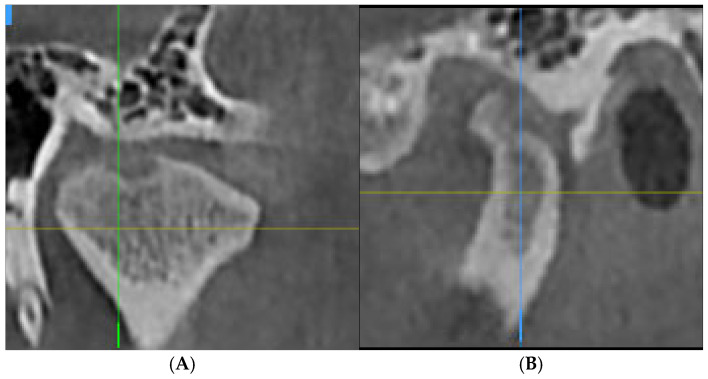
Subcortical cyst in coronal (**A**) and sagittal (**B**) sections.

**Figure 2:**
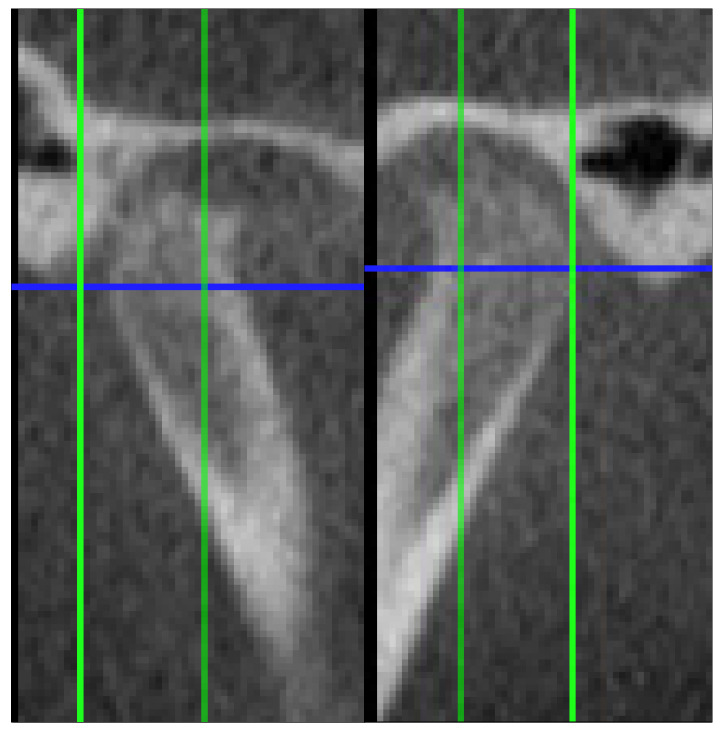
Surface erosion in sagittal section.

**Figure 3:**
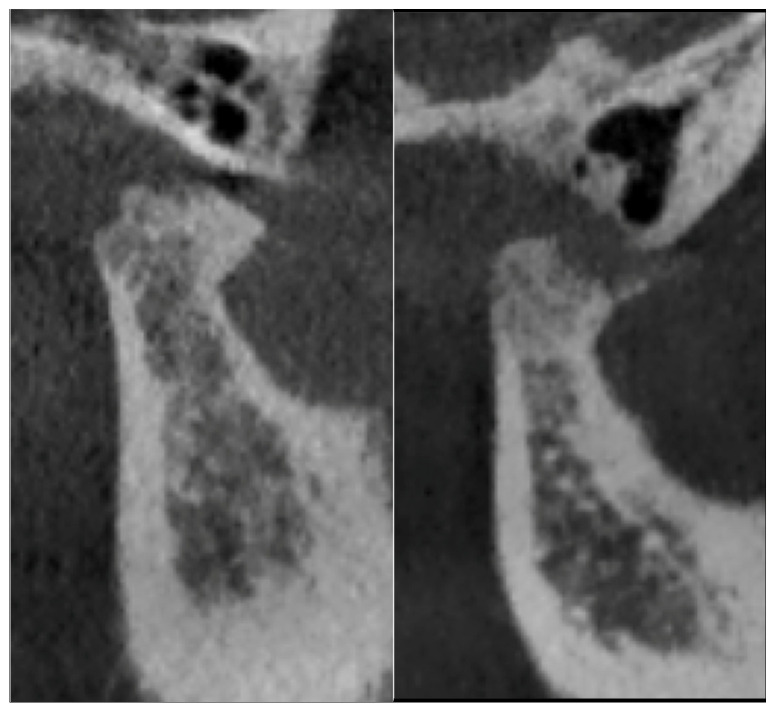
Osteophyte formation in sagittal section.

The criteria used in describing the types of degenerative alterations identified by the radiologist were derived from the RDC/TMD guidelines [22]. Degenerations such as osteophyte formation, erosion, flattening of the condyle, subcortical sclerosis or subcortical cysts were found. Figure 1, Figure 2 and Figure 3 illustrate examples of lesions identified through imaging.

### 2.3. Joint Space Assessment

**Figure 4:**
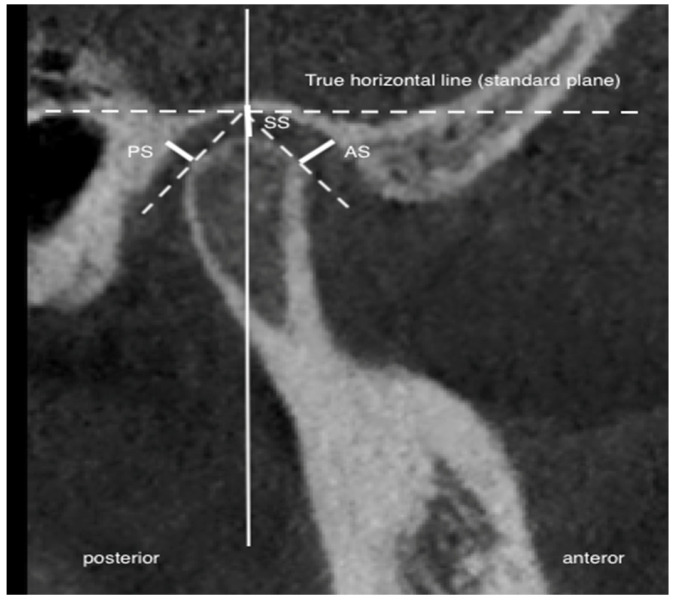
Temporomandibular joint space measurement. PS—posterior joint space; SS—superior joint space; AS—anterior joint space.

Three measurements of the joint space in the sagittal section were obtained: 1—superior joint space, from the apex of the articular head to the highest point of the articular fossa along the true horizontal line (THL) (SS); 2—anterior joint space, from the most anteriorly convex region of the articular head to the closest point of the articular fossa (AS); and 3—posterior joint space, from the most posteriorly convex region of the articular head to the closest point of the articular fossa (PS). The reference was the true horizontal line obtained from the natural head position. The linear measurements were performed following the methodology outlined by Ikeda et al. [23]. Figure 4 illustrates the previously indicated joint space measurement locations.

### 2.4. Cephalometric Analysis

Standard lateral cephalograms (Orthoralix 9200 DDE CEPH, Gendex Dental Systems, Cusano Milanino, Italy) in maximal intercuspation (MI) considering the natural head position were performed.

The cephalometric images were evaluated using the Dolphin software (version 1.8; Dolphin Imaging and Management Solutions, Chatsworth, CA, USA), a commonly used and validated tool for orthodontic and TMJ-related craniofacial analysis, by two independent examiners who were blinded to the patients’ TMJ diagnostic results, focusing specifically on vertical and horizontal discrepancies in the maxillary and mandibular positions and their interrelationships [24]. Cephalometric variables were selected based on their clinical relevance to skeletal class II analysis and whether they were affected by mandibular positional changes. Variables such as ANB, SNB, WITS, overjet, overbite, and ANS-Gn were assessed in both MIP- and centric slide-adjusted forms due to their sensitivity to mandibular displacement, as confirmed by prior studies using CR-based cephalometry [11,16]. In contrast, position-independent parameters (e.g., SNA, Ar-Go-Me), which remain stable during mandibular repositioning, were analyzed only in their original form. This approach allowed us to separate the diagnostic impact of condylar repositioning while maintaining skeletal reference consistency [21]. Cephalometric measurements are presented in Table 2.

**Figure 5:**
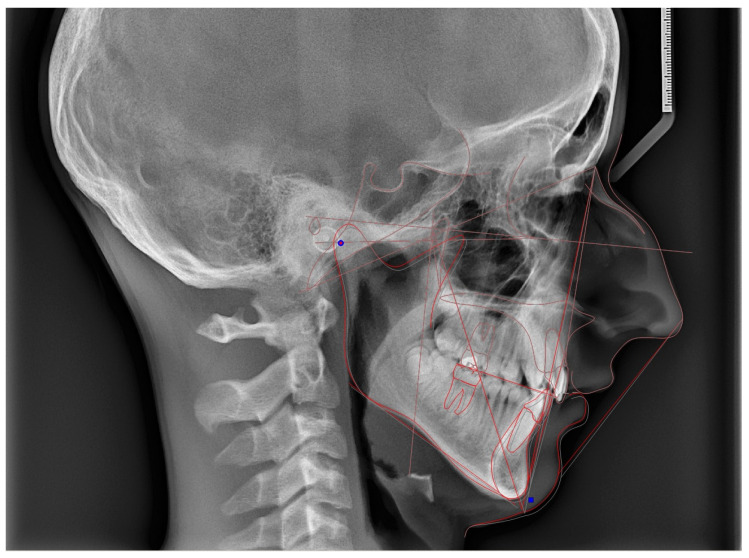
Cephalometric analysis in MIP (red lines) and after conversion to CR (grey lines).

In all patients, after identifying the cephalometric landmarks on the initial image, the transformation of the lateral cephalometric X-ray from MIP to centric relation, based on the centric slide path, was performed using computer software (Dolphin imaging software). Data from the CPI along the X- and Z-axes were used to achieve the superior–inferior and anterior–posterior alignment of the cephalometric X-ray, in accordance with the centric slide path. The effect of MIP-CR conversion on cephalometric measurements was assessed. Figure 5 visually illustrates an example of cephalometric analysis transitioning from CO to CR.

### 2.5. MIP-CR Discrepancy Assessment

CR registration was performed using Roth’s power-centric technique, a validated method for the achievement of repeatable mandibular positioning [25]. The full wax registration and mounting procedures are described in Appendix A.

Condylar displacement was defined as the positional difference (in mm) between CR and MIP, reflecting centric slide, along the anteroposterior (X), transverse (Y), and vertical (Z) axes, as recorded using a Panadent condylar position indicator (CPI), which has been shown to enable the accurate three-dimensional assessment of the condylar position [3]. A displacement ≥ 2 mm (X/Z) or ≥0.5 mm (Y) was considered clinically significant. Threshold values were based on criteria given by Utt et al. and Hidaka et al. [2,3].

**Figure 6:**
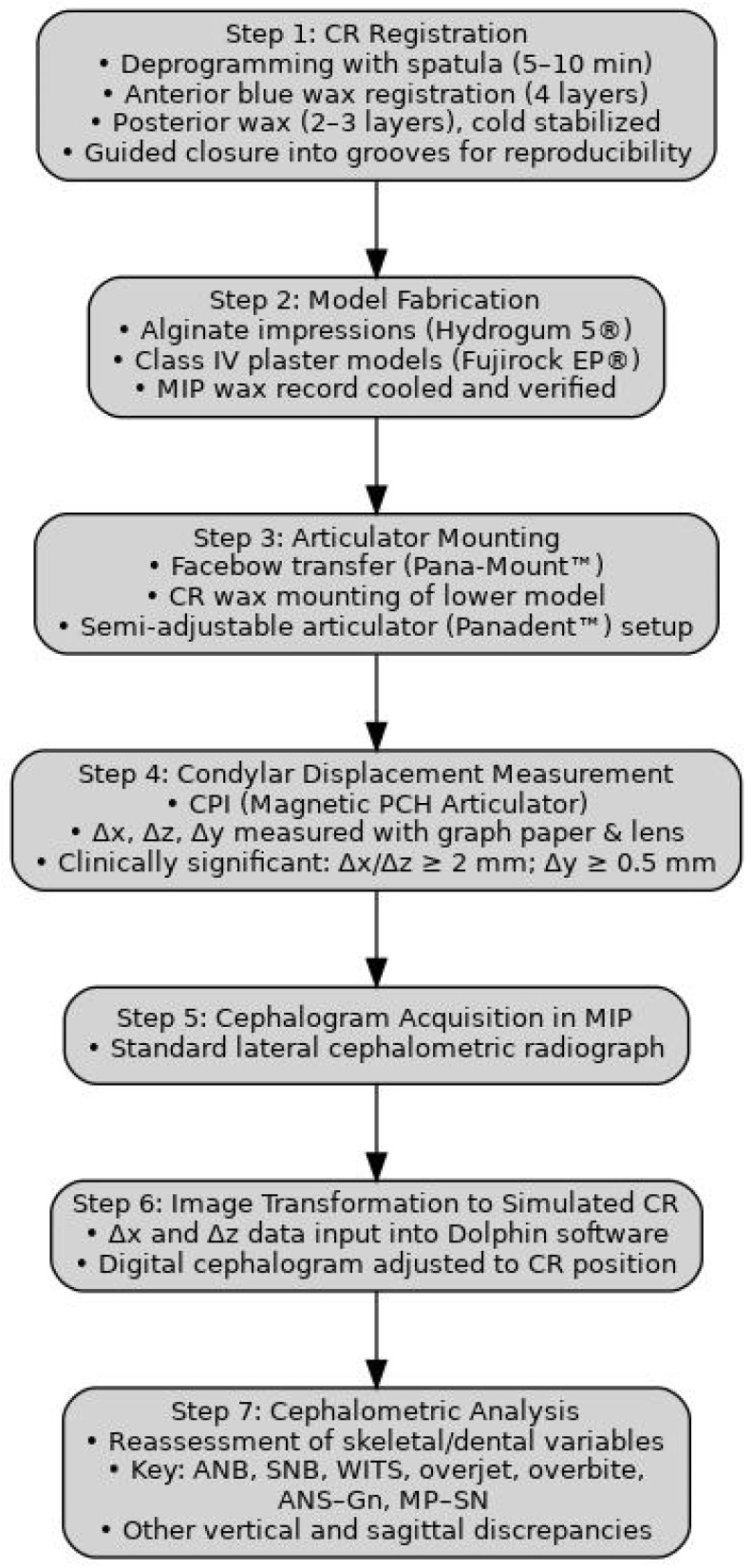
Flowchart of the CO-CR analysis protocol. The diagram outlines the workflow from clinical centric relation (CR) registration through model mounting, condylar displacement measurement using CPI, and cephalometric image adjustment in the Dolphin software. Final analysis compares cephalometric variables in maximal intercuspation (MIP) and CR to assess skeletal discrepancies in class II patients.

A simplified schematic of this process is provided in Figure 6, illustrating the workflow from CR registration to cephalometric image adjustment and CPI-guided measurement.

The reproducibility of CR registrations (10 patients, repeated after 4 weeks) was analyzed and is reported in the Section 3.

### 2.6. Measurement Reliability

To reduce observer bias, both the CBCT measurements and the condylar position assessments with CPI were conducted separately by two qualified examiners who were blinded to the patients’ group assignments. All picture files and datasets were anonymized prior to processing.

For the radiologic interpretation of osseous degenerative changes, the two examiners independently evaluated all CBCT scans according to the RDC/TMD imaging criteria. Discrepancies were resolved by consensus. The interrater agreement for the identification of osseous degenerative changes was evaluated using Cohen’s kappa coefficient (κ = 0.87), indicating strong agreement.

To verify the measurement consistency, intra- and interexaminer reliability evaluations were performed, with the aforementioned two experienced examiners independently analyzing a random subsample of 20 CBCT and cephalometric records, constituting 33% of the sample. Intraclass correlation coefficients (ICCs) were computed for essential measurements, including the joint space dimensions (anterior, superior, posterior), condylar displacement (Δx, Δy, Δz), and cephalometric parameters (ANB, SNB, overjet, Wits). The intraexaminer ICC values varied from 0.88 to 0.96, and the interexaminer ICCs ranged from 0.84 to 0.94, demonstrating substantial dependability. All measures were replicated after a two-week gap to evaluate consistency.

### 2.7. Statistical Analysis

Statistical calculations were carried out using GraphPad Prism 10.4.1 for macOS (GraphPad Software, La Jolla, CA, USA). Normality of distribution was checked using the Shapiro–Wilk test, and homogeneity of variation was checked using the Levene test. Parametric tests were used for normal distributions of quantitative data, and non-parametric tests were used in the absence of normal distributions. Comparisons between the study and control groups were performed using Student’s *t*-test and the Mann–Whitney U test. Relationships between pairs of quantitative variables were assessed using Spearman’s non-parametric correlation coefficients. Results were considered statistically significant when *p* < 0.05. Due to the number of statistical comparisons conducted, the Benjamini–Hochberg procedure was applied to control for the false discovery rate (FDR) and limit the risk of Type I error inflation. Adjusted *p*-values were reported where applicable, and statistical significance was interpreted with caution in the context of multiple testing. Between-group comparisons of age and gender revealed no statistically significant differences, confirming their status as non-confounding covariates.

## 3. Results

### 3.1. Study Population and Methodological Considerations

#### 3.1.1. Participant Characteristics

A total of 60 adult patients were included in the study, with 30 individuals in each group (control and DJD). The median age was 32 years (range: 18–52), with a female-to-male ratio of 2:1. There were no significant differences in age (*p* = 0.57) or gender distribution (*p* = 0.41) between groups, and neither variable showed a significant correlation with condylar displacement or joint space measurements.

Cephalometric analysis was conducted by two orthodontic specialists possessing over five years of professional experience. The measurements were verified for consistency, with only minor deviations noted for individual variables and the agreement levels for all tested points exceeding 85%. To enhance the credibility of the study’s findings, the analyses conducted by the two researchers were averaged.

#### 3.1.2. Reproducibility of Centric Relation Recordings

The reproducibility of the CR recordings was evaluated in a random subset of 10 patients, with repeated registrations performed after four weeks. No statistically significant differences were found between the initial and repeated measurements in any axis (X, Y, or Z), confirming the consistency of the CR registration technique (*p* > 0.05).

#### 3.1.3. Structure of the Results and Analytical Framework

To improve the clarity, Section 3 is structured to guide the reader through the most clinically relevant comparisons. First, intergroup differences were analyzed to identify skeletal and condylar characteristics that distinguished the study and control groups, including the role of condylar displacement. These comparisons emphasized discrepancies in the cephalometric measurements and condyle position between groups. Second, intragroup analyses of CO-CR conversion were performed to assess the impact of centric relation adjustment on cephalometric variables, with particular attention to patients in the study group presenting condylar displacement ≥ 2 mm. Third, correlation analyses were conducted to explore associations between condylar displacement, joint space dimensions, and cephalometric variables in order to identify the most clinically significant patterns. Throughout this section, special emphasis is placed on sagittal discrepancies, which emerged as the dominant feature in this study population.

### 3.2. Intergroup Differences

#### 3.2.1. Condylar Bone Change Prevalence

In the study group, degenerative bone alterations observed on CBCT included the following:

- Osteophyte formation: 22/60 cases (36.7%);
- Erosion: 18/60 cases (30.0%);
- Subcortical sclerosis: 10/60 cases (16.7%);
- Articular surface flattening: 6/60 cases (10.0%);
- Subcortical cysts: 4/60 cases (6.7%).

The results demonstrate that osteophytes and erosions are the predominant radiological indicators of degenerative joint disease in people with class II malocclusion.

Detailed findings are elaborated in Table S1 of the Supplementary Tables.

#### 3.2.2. Cephalometric Variables

**Figure 7:**
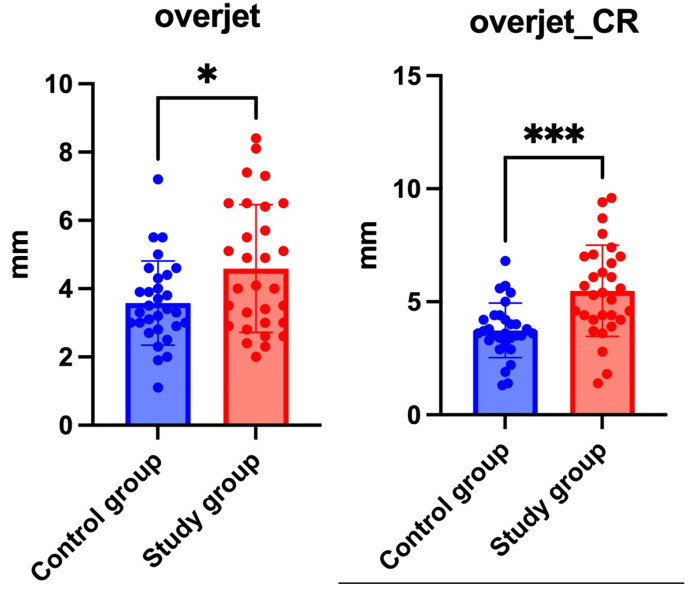
Distribution of overjet measurements in control and study groups, presented in both maximal intercuspation (MIP) and centric relation (CR). Significantly greater overjet was observed in the study group compared to controls, with the difference further increasing after CR adjustment (* indicates *p* < 0.05 in MIP; *** indicates *p* < 0.001 in CR).

The study group exhibited significantly greater overjet values than the controls in both MIP (4.6 ± 1.9 mm vs. 3.6 ± 1.2 mm; *p =* 0.0164) and CR-adjusted conditions (5.5 ± 2.0 mm vs. 3.7 ± 1.2 mm; *p* < 0.0001) as shown in Figure 7. Importantly, the mean difference of 1.8 mm in centric slide-adjusted overjet indicates that the skeletal discrepancies may be even more extensive than initially presumed, especially in patients with temporomandibular joint degeneration, where an altered condylar morphology may contribute to occlusal instability. This reinforces the clinical relevance of overjet as a sensitive indicator of anteroposterior malocclusion, particularly when assessed in centric relation (via centric slide adjustment), highlighting the diagnostic value of CR analysis.

Similarly, sagittal variables such as ANB and WITS showed more pronounced discrepancies between groups in CR, which suggests that CR-based evaluation may enhance the detection of true skeletal class II characteristics by minimizing the masking effects of occlusal compensation.

Table 3 summarizes the main findings. Complete data can be found in Table S2 of the Supplementary Tables.

#### 3.2.3. Joint Space

**Figure 8:**
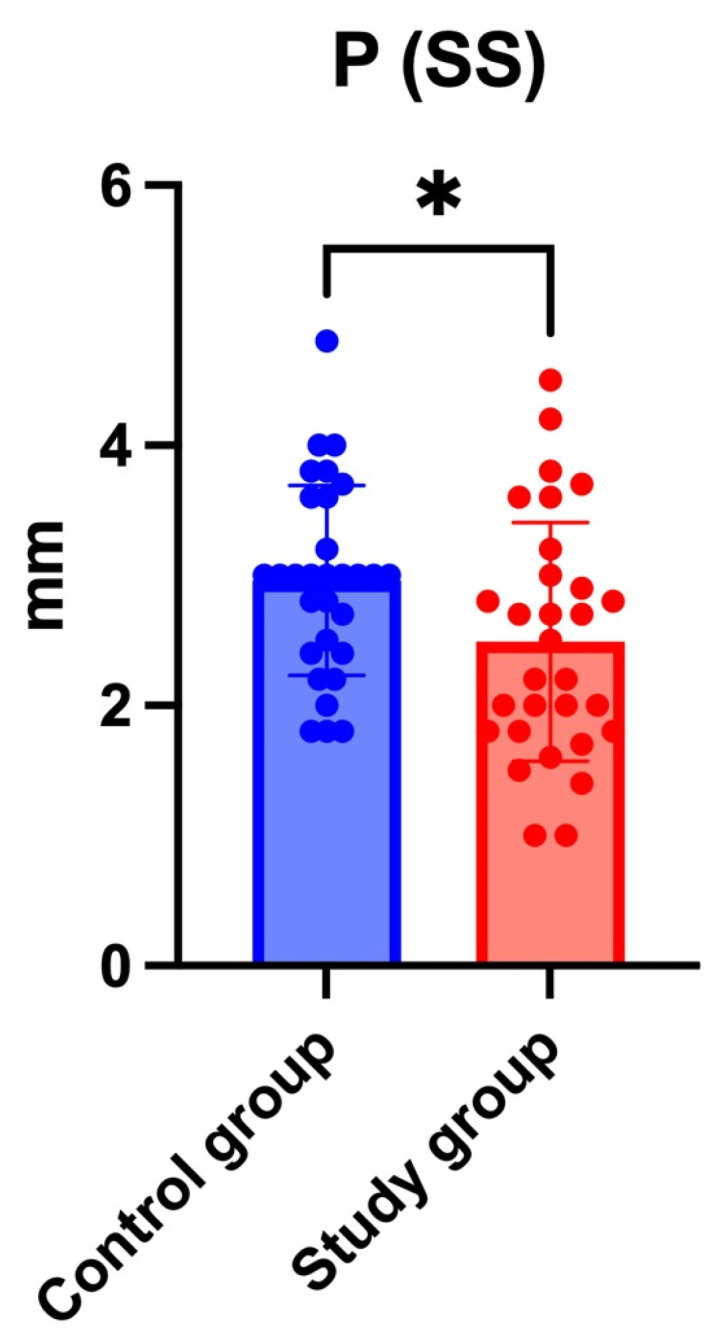
Distribution of superior joint space (SS) measurements in the right temporomandibular joint (TMJ) for the control and study groups. A statistically significant reduction (* indicates *p* < 0.05) in the superior joint space was observed in the study group compared to controls (right TMJ, *p* = 0.031).

Statistical analysis using a *t*-test revealed no significant differences in the AS, SS, and PS values among the groups, except for the superior joint space in the right joint with *p* = 0.031 (as shown in Figure 8). The statistically significant narrowing of the superior joint space in the right TMJ may suggest early degenerative remodeling, potentially relevant for treatment planning. The mean joint space measurements for both groups on the left and right sides are available in the Supplementary Tables under Table S3.

#### 3.2.4. Condylar Displacement

Condylar displacement between maximal intercuspation and centric relation was evaluated in three dimensions. No statistically significant differences were found between groups in the anteroposterior (Δx), vertical (Δz), or transverse (Δy) dimensions. However, a trend toward increased Δz (vertical displacement) in the right joint was observed in the study group. The results for both groups can be found in the Supplementary Tables under Table S4.

Clinically relevant displacement (Δx or Δz ≥ 2 mm):

- Study group: 54/120 condyles (45%);
- Control group: 29/120 condyles (24%).

Vertical displacement (Δz ≥ 2 mm):

- Study group: 27 condyles (22.5%);
- Control group: 19 condyles (15.8%).

Transverse displacement (Δy ≥ 0.5 mm):

- Study group: 16 patients (26.7%);
- Control group: 9 patients (15.0%).

**Figure 9:**
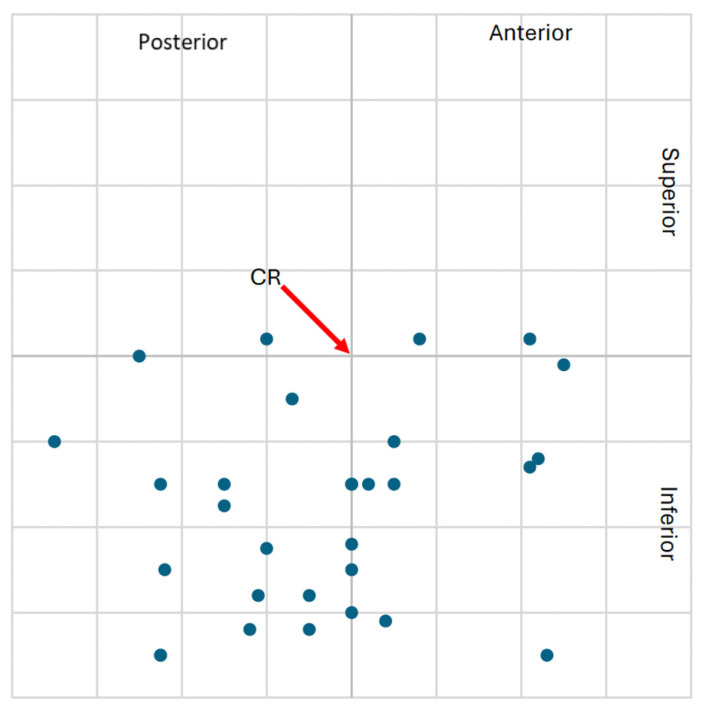
Graphical representation of the left condylar process positions (n = 30) in the study group, illustrating displacements (centric slide) from maximal intercuspation (MIP) to centric relation (CR) on a 1 mm coordinate grid. Most condyles exhibited downward and anterior or posterior shifts relative to CR.

**Figure 10:**
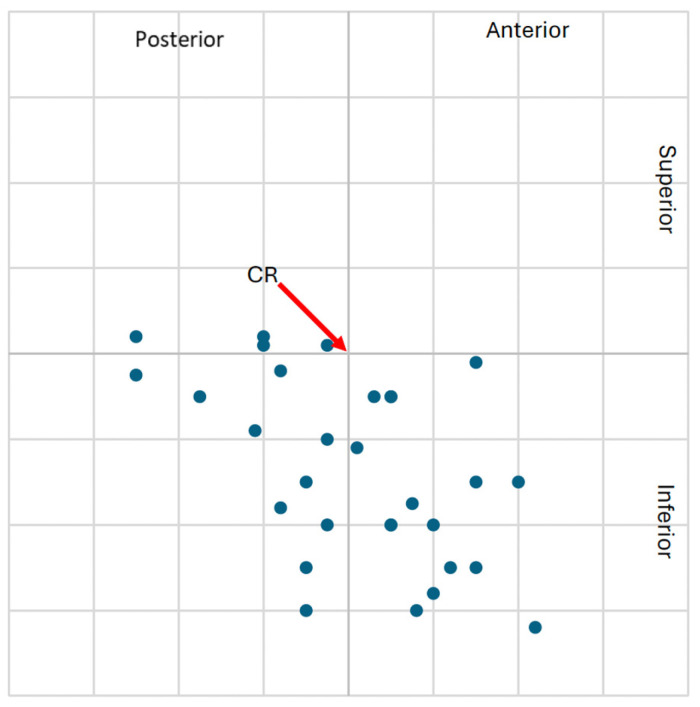
Graphical representation of the right condylar process positions (n = 30) in the study group, illustrating displacements (centric slide) from maximal intercuspation (MIP) to centric relation (CR) on a 1 mm coordinate grid. Condyles predominantly shifted downward and anteriorly or posteriorly in relation to CR.

**Figure 11:**
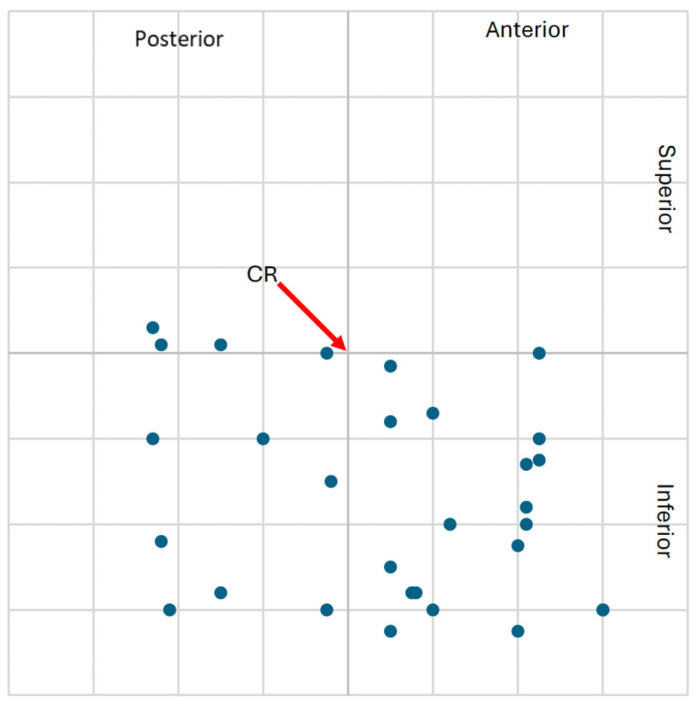
Graphical representation of the left condylar process positions (n = 30) in the control group, illustrating displacements (centric slide) from maximal intercuspation (MIP) to centric relation (CR) on a 1 mm coordinate grid. Most condylar shifts presents fewer clinically significant displacements compared to the study group.

**Figure 12:**
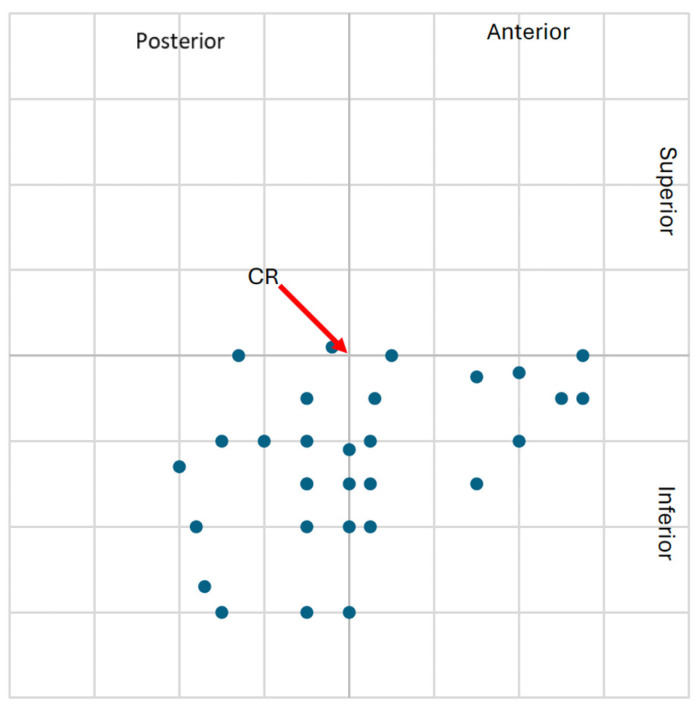
Graphical representation of the right condylar process positions (n = 30) in the control group, illustrating displacements (centric slide) from maximal intercuspation (MIP) to centric relation (CR) on a 1 mm coordinate grid. The majority of condylar shifts were restricted in extent, with few occurrences of clinically relevant displacement.

The higher frequency of clinically significant condylar displacements in the study group supports the hypothesis that class II malocclusion with TMJ degeneration is associated with altered mandibular positioning. These spatial displacements are visualized in Figure 9, Figure 10, Figure 11 and Figure 12, showing the condylar position on a 1 mm grid in both groups.

### 3.3. Impact of Centric Slider Conversion on Cephalometric Variables

#### 3.3.1. Study Group

The comparison of the cephalometric characteristics before and after conversion to centric relation using articulator data revealed no statistically significant differences, except for overbite, which significantly decreased after mandibular repositioning via centric slide from MIP to CR (mean change: 1.38 ± 0.56 mm; *p* = 0.016). Although the *p*-value indicates statistical significance, the clinical magnitude of this change was modest. The mean change of 1.38 mm may have resulted from the minor vertical repositioning of the condyles, neuromuscular adaptation, or soft tissue compression during wax registration. Therefore, this difference should be interpreted with caution, as it may reflect methodological variations rather than a true anatomical change. Nevertheless, the consistent direction of change supports the notion that vertical interarch relationships may be underestimated in maximal intercuspation.

A trend toward significance was observed for overjet (*p* = 0.08), suggesting that the centric relation adjustment of the mandible may reveal hidden occlusal discrepancies, particularly in vertical and sagittal overlap. However, given the lack of statistical significance and possible variability due to the registration technique, this conclusion requires careful interpretation.

The differences between the means and *p*-values in the cephalometric measurement comparison according to condylar displacement can be found in Table S5 in the Supplementary Tables.

#### 3.3.2. Control Group

No statistically significant changes were observed in the control group when the cephalometric data were converted from maximal intercuspation to centric relation. This supports the assumption that stable TMJs without degenerative alterations tend to show minimal shifts in skeletal and dental measurements following CR repositioning. The variations in the means and *p*-values in the comparison of the cephalometric measurements based on condylar displacement are summarized in Table S6 in the Supplementary Tables.

#### 3.3.3. Study Group with CD ≥ 2 mm

Patients with clinically relevant condylar displacement (Δx or Δz ≥ 2 mm) were compared within the study group to assess its effect on cephalometric outcomes. The following key findings were noted.

- Significant differences in ANB:
  - Left condyle Δx ≥ 2 mm → *p* = 0.006;
  - Right condyle Δ x ≥ 2 mm → *p* = 0.05.
- Highly significant differences in overjet:
  - Both condyles Δ x ≥ 2 mm → *p* = 0.0002;
  - Δz ≥ 2 mm → *p* = 0.001 (left), *p* = 0.007 (right).
- Trend for WITS difference (left condyle Δx): *p* = 0.08.
- Trend in ANS-Gn distance (left condyle Δz): *p* = 0.08.

In patients with condylar displacement ≥ 2 mm, particularly along the sagittal axis (Δx), CR-based overjet and ANB were consistently higher. For example, in the left condyle subgroup with Δx ≥ 2 mm, mean overjet_CR increased by more than 2 mm (*p* = 0.0002), reinforcing the clinical impact of condylar displacement on sagittal discrepancies. In contrast, vertical parameters showed less consistent variation, indicating that the primary effect of condylar shift involves anteroposterior relationships. A summary of these correlations is available in Table 4 and Supplementary Table S7.

### 3.4. Correlations of Variables

#### 3.4.1. Condylar Displacement and Cephalometric Variables

In the study group, moderate to substantial associations were noted between condylar displacements, particularly in the anteroposterior (Δx) and vertical (Δz) axes, and several sagittal and vertical cephalometric parameters. The most notable finding was the correlation between Δx of the right condyle and ANB (r = 0.46; *p* = 0.012), ANS-Me (r = 0.5; *p* = 0.005), and ANS-Gn in CR (r = 0.44; *p* = 0.15), indicating that greater posterior displacement may be linked to exaggerated skeletal class II malocclusion. In contrast, vertical displacement (Δz) showed a more limited relationship, with a moderate correlation observed only between the left-side Δz and ANS-Me in CR (r = −0.36; *p* = 0.049), indicating a potential association with vertical mandibular positioning in selected cases.

Δx on the left condyle also showed associations with the mandibular length and inclination, including the Co-Gn angle. However, this correlation was moderate in strength, and their interpretation should be approached with caution due to potential confounding factors such as facial growth patterns and measurement sensitivity.

Significantly, these relationships were either weak or not observed in the control group, underscoring the notion that clinically significant condylar displacements (≥2 mm) may indicate an underlying skeletal discrepancy in individuals with TMJ degenerative changes. A overview of the notable associations for both groups may be seen in Supplementary Tables S8 and S9.

#### 3.4.2. Joint Space and Cephalometric Variables

Our research indicates that varying joint space widths may correlate with increasing or decreasing values of particular cephalometric variables. Several joint space dimensions—particularly the superior and posterior joint space (SS, PS)—showed significant correlations with vertical skeletal variables, such as MP/SN, FMA, and the Go-Gn angles, within the study group.

For example, the right PS showed a statistically significant correlation with MP/SN (r = −0.43; *p* = 0.016), which may reflect the adaptive vertical repositioning of the condyle in patients with posterior joint space narrowing.

Additionally, a significant correlation was found between vertical condylar displacement (Δz) and the posterior joint space (r = 0.41; *p* = 0.025) in the right TMJ. While this association may point to altered joint loading and remodeling, the moderate strength of the correlation suggests that it may also be influenced by individual anatomical variability. Such correlations were absent in the control group, further supporting the idea that the joint space alterations in the study group reflect compensatory or degenerative adaptations.

The detailed correlation matrix is included in Supplementary Table S10.

## 4. Discussion

This study aimed to assess the impact of the maxillofacial morphology and condylar displacement on degenerative alterations in the temporomandibular joint (TMJ) among adult patients with skeletal class II malocclusion. This work provides novel insights into the correlation between skeletal patterns and radiographic modifications of the TMJ by integrating three-dimensional CBCT imaging and cephalometric evaluation in both the MI and CR positions. Our findings are consistent with previous studies demonstrating associations between skeletal patterns and TMJ pathologies, although causal interpretations must be approached cautiously due to the cross-sectional nature of the design [13,14,26].

### 4.1. Methodological Reliability and Centric Relation Reproducibility

The high reproducibility of centric relation positioning across the X-, Y-, and Z-axes observed in our study supports the validity of our protocol. The over 85% interexaminer agreement in cephalometric tracing further strengthens the methodological consistency. However, formal calibration procedures between observers were not performed, representing a limitation of the study. Prior studies have emphasized the significance of CR reproducibility for reliable TMJ assessments and orthodontic diagnostics [12,27,28].

### 4.2. Prevalence and Patterns of Condylar Bone Alterations

Osteophyte formation (36.7%) and erosion (30%) were the most common CBCT findings in the study group, followed by sclerosis, flattening, and subcortical cysts. This distribution differs from reports such as Oliveira et al., where flattening was more prevalent. Such differences may reflect variations in the CBCT resolution, diagnostic thresholds, or population characteristics. Osteophyte formation may indicate an adaptive response to altered joint loading, as proposed by Tanaka et al. and Paknahad et al. [4,12,28].

### 4.3. Differences in Cephalometric Variables

Significantly increased overjet was observed in the study group, both in MIP and CR (*p* = 0.016; *p* = 0.0001), indicating a pronounced skeletal sagittal discrepancy. Although the ANB angle after centric slide adjustment approached significance (*p* = 0.055), this trend aligns with earlier studies by Shi et al. and Chen et al., which report that CR-based assessments can reveal skeletal discrepancies otherwise concealed in habitual occlusion [11,16]. Notably, these changes were observed only in the study group and may reflect condylar remodeling or altered joint loading affecting the mandibular position. In contrast, the control group exhibited less pronounced changes between positions, indicating a more stable condylar and occlusal relationship.

### 4.4. Temporomandibular Joint Space Variation

Among all the joint space measurements, only the superior joint space on the right side showed a significant reduction (*p* = 0.031). This localized reduction may reflect incipient degenerative remodeling, as suggested in the study by Zhou et al. [29]. However, isolated joint space changes without broader structural evidence may not suffice to confirm degenerative progression, highlighting the importance of correlating imaging with clinical function.

### 4.5. Condylar Displacement Due to Centric Slide Between Groups

Although the raw condylar displacement values did not differ significantly between the study and control groups, clinically relevant displacements (≥2 mm) were notably more frequent in the study group (45% vs. 24%). This suggests that patients with TMJ alterations may demonstrate greater variability in mandibular positioning. These observations are consistent with findings obtained by Alhammadi et al. and Muthuraman et al., who reported increased condylar asymmetry in class II malocclusion. These centric slide displacements may contribute to uneven joint loading, potentially increasing the susceptibility to internal derangement, as proposed by Muthuraman et al. and Das et al. [19,30,31]. However, it is important to note that displacement alone does not confirm dysfunction and should be evaluated alongside clinical signs and detailed radiographic analysis.

### 4.6. Impact of MIP-CR Conversion on Cephalometric Variables

#### 4.6.1. Group Comparison

Following CR repositioning, the study group showed a significant increase in overjet and a reduction in overbite. While these findings suggest that MIP-CR discrepancies may influence cephalometric interpretation, especially in the sagittal plane, the magnitude of the vertical changes was modest and may reflect registration variability or soft tissue compression. Previous investigations (by Shi et al. and Krisjane et al.) have shown similar trends, reinforcing the diagnostic value of CR-based cephalometry in class II malocclusion [7,16].

#### 4.6.2. Study Group with CD ≥ 2

In patients with condylar displacement ≥ 2 mm, the CR-adjusted overjet and ANB were significantly greater, indicating that condylar translation may exaggerate class II skeletal features. This group-specific effect was not seen in the controls and suggests that condylar shifts in DJD patients may contribute to altered skeletal diagnoses. While these findings are in line with observations by Alhammadi et al. and Muthuraman et al., they should be interpreted as correlational rather than causal and considered alongside clinical examination [30,31]. These findings emphasize that CR-based diagnostics can detect subtle but clinically relevant discrepancies that may otherwise go unnoticed, warranting caution when determining treatment mechanics and retention planning.

### 4.7. Correlations of Variables

#### 4.7.1. Condylar Displacement and Cephalometric Variables

Moderate correlations were observed between Δx and sagittal cephalometric parameters (ANB, ANS-Gn), as well as between Δz and vertical dimensions. These associations were present in the study group but not in the controls, suggesting a relationship between the condylar position and craniofacial morphology. However, the moderate strength of these correlations indicates that other factors, such as growth patterns or joint adaptation, may also be involved [21,32].

#### 4.7.2. Joint Space and Cephalometric Variables

The superior and posterior joint spaces showed moderate correlations with vertical skeletal measurements, such as FMA and SN/Go-Gn, particularly in the study group. These findings are consistent with prior work by Al-Hadad et al. and Choi et al. and may reflect compensatory changes in hyperdivergent patterns. No such associations were seen in the control group, supporting the idea that joint remodeling contributes to occlusal variation in class II patients [21,33].

### 4.8. Strengths and Limitations

This study benefits from a homogeneous adult sample, standardized imaging protocols, and the integration of CR and cephalometric parameters. However, limitations include the cross-sectional design, lack of longitudinal follow-up, modest sample size due to strict inclusion criteria, lack of interrater calibration, and potential observer-related bias. Scanner variability and patient positioning during CBCT acquisition may also influence the measurement reliability, particularly in vertical landmarks. While the study highlights important associations between TMJ degeneration and skeletal discrepancy, causality cannot be inferred. Previous systematic reviews have highlighted the complexity of the relationship between skeletal malocclusion and TMJ alterations [13,34]. Future prospective studies with larger cohorts are needed to clarify these interactions, confirm potential causal links, and explore the progression of TMJ changes over time.

### 4.9. Future Directions

We recommend that future research should

1. Incorporate longitudinal designs to evaluate TMJ remodeling over time;
2. Investigate the prognostic value of MIP-CR discrepancies in detecting TMJ dysfunction;
3. Establish standardized CBCT and CR recording protocols for routine orthodontic screening in high-risk populations.

## 5. Conclusions

Based on the results obtained in this study, the following detailed conclusions can be drawn:

1. Adult patients with skeletal class II malocclusion demonstrated a higher prevalence of CBCT-confirmed temporomandibular joint degeneration.
2. Greater condylar displacements between CR and MIP were observed in the DJD group, particularly in the anteroposterior (Δx) and vertical (Δz) dimensions.
3. Cephalometric differences, including increased ANB, reduced SNB, and higher WITS values, indicated more pronounced sagittal discrepancies in the study group.
4. Posterior and superior joint space reductions may reflect condylar remodeling or altered joint loading in the degenerative group.
5. These findings suggest that CR-based assessment and condylar position analysis may enhance the understanding of skeletal and joint-related discrepancies in selected adult cases. However, clinical implementation should be approached cautiously and supported by further prospective validation.
6. Due to the cross-sectional design and moderate sample size, these observations should be interpreted cautiously. Longitudinal studies are warranted to confirm these associations and determine their diagnostic and prognostic value.

## Figures and Tables

**Table 1:** Inclusion and exclusion criteria.

Inclusion Criteria	Exclusion Criteria
Skeletal class II malocclusion (ANB angle values above the 2 ± 2.5° interval) Age 18 years or older TMJ sounds and mild-to-moderate TMJ pain No prior orthodontic treatment	Congenital craniofacial malformations Severe facial asymmetry Deformity secondary to trauma Ankylosis Endocrine–metabolic diseases/severe systematic diseases Autoimmune diseases Rheumatoid/other type of arthritis Unilateral condylar bony changes Acute or severe TMJ pain, masticatory muscle tenderness, or a restricted range of mandibular motion

**Table 2:** Angular and linear cephalometric variables.

Angular Cephalometric Variables	Description	Linear Cephalometric Variables	Description
SNA SNB ANB PP/MP PP/Go-Gn Ar/Go-Me PP/SN SN/Go-Gn FMA MP/SN occ/SN	sella–nasion–point A sella–nasion–point B difference between SNA and SNB palatal plane/mandibular plane palatal plane/gonion–gnathion articulare/gonion–menton palatal plane/sella–nasion sella–nasion/gonion–gnathion Frankfurt mandibular plane angle mandibular plane/sella–nasion occlusal plane/sella–nasion	WITS ANS-Gn ANS-Me Co-Gn/Co-A overjet overbite	Wits appraisal (points A and B relation on occlusal plane) anterior nasal spine–gnathion (lower face height) anterior nasal spine–menton condylion–gonion/condylion–point A horizontal overlap of the maxillary central incisors over the mandibular central incisors vertical overlap of the maxillary central incisors over the mandibular central incisors

**Table 3:** Results of cephalometric linear and angular measurements.

Cephalometric Variable	Study Group (Mean ± SD)	Control Group (Mean ± SD)	*p*-Value
ANB_CR (degrees)	7.4 ± 2.5	5.9 ± 1.1	0.055
Overjet (mm)	4.6 ± 1.9	3.6 ± 1.2	0.0164 *
Overjet_CR (mm)	5.5 ± 2	3.7 ± 1.2	0.0001 ***

variables that remain constant during centric slide adjustment; SD—standard deviation; * *p* < 0.05; *** *p* < 0.001; CR—Centric relation.

**Table 4:** Comparison of cephalometric linear and angular measurements in study group with CD ≥ 2 according to Δx i Δz.

Cephalometric Variable	*p*-Value (Δx L)	*p*-Value (Δx R)	*p*-Value (Δz L)	*p*-Value (Δz R)
ANB_CR	0.0057 *	0.048 *	0.3	0.26
WITS_CR	0.08	0.31	0.64	0.34
ANS-Gn_CR	0.84	0.32	0.08	0.77
Overjet_CR	0.0002 ***	0.0002 ***	0.0013 *	0.0068 *

L—left condyle; R—right condyle; Δx—anteroposterior displacement; Δz—vertical displacement; significant differences were determined at * *p* < 0.05; *** *p* < 0.001; CR—centric relation.

## Data Availability

The datasets used and/or analyzed in this study are available from the corresponding author upon reasonable request.

## Supplementary Materials

The following supporting information can be downloaded at: https://www.mdpi.com/article/10.3390/jcm14134499/s1, Figure S1. Flowchart of the CO–CR analysis protocol. The diagram outlines the workflow from clinical centric relation (CR) registration through model mounting, condylar displacement measurement using CPI, and cephalometric image adjustment in Dolphin software. Final analysis compares cephalometric variables in MIP and CR to assess skeletal discrepancies in Class II patients; Table S1. Distribution of patients on the basis of condylar osseous features. Table S2. Results of cephalometric linear and angular measurements. Table S3. Results of linear measurements of joint space in saggital plane. Table S4. Displacement of condylar processes in the maximal intercuspation position. Table S5. Results of CO-CR conversion of cephalometric linear and angular measurements in study group. Table S6. Results of CO-CR conversion of cephalometric linear and angular measurements in control group. Table S7. Comparison of cephalometric linear and angular measurements in study group with CD>=2 according to Δx i Δz. Table S8. Correlations of cephalometric linear and angular measurements as well as condylar displacement in study group with Δx i Δz. Table S9. Correlations of cephalometric linear and angular measurements as well as condylar displacement in control group with Δx i Δz. Table S10. Correlations of cephalometric linear and angular measurements as well as condylar displacement and joint space in study group.

## Abbreviations

The following abbreviations are used in this manuscript:

TMJ	temporomandibular joint
CBCT	cone beam computed tomography
CPI	condylar position index
SS	superior joint space
AS	anterior joint space
PS	posterior joint space
DJD	degenerative joint disease
AAOP	American Academy of Orofacial Pain
CR	centric relation
MIP	maximal intercuspation, habitual occlusion
CD	condylar displacement

## Appendix A

### Appendix A.1. Imaging Studies

High-resolution 3D cone beam computed tomography imaging (IS i-CAT Next Generation KaVo Imaging, Hatfield, PA, USA) was conducted with the following scanning settings of the CBCT machine: field of view: 23 cm × 17 cm (height/width), voxel size: 0.3 mm (scan duration: 17.8 seconds), 120 kVp and 37.10 mAs. The patient was seated in the natural head position, with the teeth in maximum intercuspation, the back as perpendicular to the floor as possible, and the eyes focused on a point at the same level in a mirror for imaging. The Frankfort horizontal plane of the patient was parallel to the floor.

### Appendix A.2. CO-CR Discrepancy Assessment

Alginate mass [Hydrogum 5; Zhermack S.p.A., Badia Polesine (Rovigo), Italy] was used to make impressions for the models on metal spoons with a deepened rim (Algilock®; Hager&Werken GmbH&Co. KG, Duisburg, Germany). Advanced orthodontic diagnostic models fabricated from class IV high-strength dental synthetic plaster (Fujirock EP®; GC EUROPE N.V., Leuven, Belgium) were performed. The assessment of models in MI was performed using an intraoral record made of pink soft modeling wax (Modeling wax; Zhermack Sp. z o.o., Warsaw, Poland), which the patient was directed to bite down in the maximum intercuspation position. The accuracy of the retrieved record was re-evaluated in the patient’s mouth following immersion in ice-cold water. Roth’s “power centric” method was used to determine the centric relation (CR) following neuromuscular deprogramming, which involved pulsatile biting of a wooden spatula for five to ten minutes. The CR position was then registered with the blue wax (Bite Registration Sheet Wax, Almore International, Inc., Portland, OR, USA) in two parts. After placing the patient at a 45˚ angle to the ground and heating the wax in a water bath (06-DK-2000-1; “CHEMLAND” Technical and Commercial Company, Stargard, Poland) to a temperature of 57˚, a four-layer anterior wax register was made. The operator directed the patient’s mandible during mouth closure to prevent protrusion. This method was described by Wood et al. (Wood and Elliott 1994) [1].

The anterior register was subsequently cooled for 5 minutes in ice-cold water and positioned between the patient’s dental arches alongside a warmed posterior register composed of two or three layers. The operator first directed the patient’s jaw, then told the patient to bite down firmly when reaching the correct grooves in the anterior register. The models in CR were analyzed using a Panadent® articulator. The registration of the jaw position utilizing a face-bow (The Pana-Mount ™ Face-Bow, 580 S. Rancho Ave. Colton, CA 92324, USA) facilitated the mounting of the upper model in the articulator with articulating plaster (Stodent III arti; Zhermack Sp. z o.o., Warsaw, Poland), as well as CR registers were employed to mount the lower model. To assess the reproducibility of CR registrations, 10 patients were randomly chosen, and CR registrations were conducted again after a period of 4 weeks. New mandibular models were fitted to pre-mounted maxillary models in these patients, and the results of both registrations were utilized for a comparison procedure.

Condylar displacement (CD) on the right and left side was quantified on the models utilizing a condylar position indicator (CPI) with a gauge (Magnetic PCH Articulator, 580 S. Rancho Ave., Colton, CA 92324, USA) across three-dimensional planes, evaluating the position of the condylar processes in maximum intercuspation (MI) in relation to the hinge axis of the articulator, which signifies centric relation (CR). The CR-MI difference was assessed along the anteroposterior (X), vertical (Z), and transverse (Y) axes. The Δx and Δz measurements were conducted on millimeter graph paper utilizing a magnifying lens with 0.1mm graduations. Each measurement was conducted twice by the primary researcher.

Criteria described by Uttet al. (Utt, Meyers et al. 1995) [2] and Hidaka et al. (Hidaka, Adachi et al. 2002) [3] were used to evaluate the position of the condyle. Values of x < 1, z < 1, y < 0.5 were defined as the ideal position range of the mandibular condylar process. A discrepancy of 2 mm or more in the anteroposterior and vertical axes or 0.5 mm or more in the transverse direction was considered clinically significant.
