# Supplementary material for: Influence of Maxillofacial Morphology on Temporomandibular Joint Degenerative Alterations and Condyle Position Assessed by CBCT in Class II Malocclusion Adult Patients—A Cross-Sectional Study
Source: J Clin Med. 2025 Jun 25;14(13):4499. doi: 10.3390/jcm14134499 (PMC12249668; doi:10.3390/jcm14134499)
Supplement: Supplementary file 1 [file jcm-14-04499-s001.zip › Flowchart .pdf]

Step 1: CR Registration
│
├── Neuromuscular deprogramming using spatula (5–10 minutes)
├── Anterior CR registration with heated blue wax (4 layers)
├── Posterior wax registration (2–3 layers), cold stabilized
└── Jaw guided closure into wax grooves to ensure reproducibility
↓
Step 2: Model Fabrication
│
├── Alginate impressions (Hydrogum 5®)
├── Models poured in Class IV high-strength plaster (Fujirock EP®)
└── MIP record taken with soft modeling wax, cooled and verified
↓
Step 3: Articulator Mounting
│
├── Pana-Mount™ facebow used for upper model transfer
├── CR wax record used to mount mandibular model
└── Semi-adjustable articulator (Panadent™) setup with articulating plaster
↓
Step 4: Condylar Displacement Measurement
│
├── CPI (Magnetic PCH Articulator) used to assess condylar position
├── Δx (anteroposterior), Δz (vertical), Δy (transverse) measured
├── Graph paper and magnifying lens (0.1 mm precision) used
└── Clinically significant: Δx or Δz ≥ 2 mm; Δy ≥ 0.5 mm
↓
Step 5: Cephalogram Acquisition in MIP
│
└── Standard lateral cephalometric radiograph taken in MIP
↓
Step 6: Image Transformation to Simulated CR
│
├── Δx and Δz data input into Dolphin software
└── Cephalometric x-ray digitally adjusted to simulate CR position
↓
Step 7: Cephalometric Analysis
│
├── Reassessment of skeletal and dental measurements on CR image
└── Key variables: ANB, SNB, WITS, overjet, overbite, ANS–Gn, MP–SN
└── other cephalometric variables: vertical and sagittal
↓
Step 8: CR vs MIP values comparison

Figure S1. Flowchart of the CO–CR analysis protocol. The diagram outlines the workflow from clinical centric relation (CR) registration through model mounting, condylar displacement measurement using CPI, and cephalometric image adjustment in Dolphin software. Final analysis compares cephalometric variables in MIP and CR to assess skeletal discrepancies in Class II patients.
