# Supplementary material for: Influence of Maxillofacial Morphology on Temporomandibular Joint Degenerative Alterations and Condyle Position Assessed by CBCT in Class II Malocclusion Adult Patients—A Cross-Sectional Study
Source: J Clin Med. 2025 Jun 25;14(13):4499. doi: 10.3390/jcm14134499 (PMC12249668; doi:10.3390/jcm14134499)
Supplement: Supplementary file 1 [file jcm-14-04499-s001.zip › Supplementary Tables.pdf]

Table S1. Distribution of patients on the basis of condylar osseous features.

Type of degeneration	N	%
osteophyte	22	36,7
erosion	18	18
subcortical sclerosis	10	10
articular surface flattening	6	6
subcortical cysts	6	4

Table S2. Results of cephalometric linear and angular measurements.

Cephalometric variable	study group mean SD	control group mean SD	p-value
**SNA~ (degrees)**	82.8 3.9	81.8 3.5	0.32
SNB (degrees)	77 4	77.2 3.6	0.99
SNB_CR (degrees)	76 3.7	76.9 3.5	0.65
ANB (degrees)	6.2 1.7	5.7 1.2	0.23
ANB_CR (degrees)	7.4 2.5	5.9 1.1	0.055
**PP/MP~ (degrees)**	27.4 6.8	25.6 4.5	0.23
**PP/Go-Gn~ (degrees)**	24.1 6.9	22.2 5.4	0.24
**Ar/Go-Me~ (degrees)**	121.9 8.6	121.1 6.3	0.68
**PP/SN~ (degrees)**	7.1 3.1	7.4 3.5	0.67
**SN/Go-Gn~ (degrees)**	32 7.4	30.5 5.7	0.36
**FMA~ (degrees)**	25.6 8.4	23.7 5.7	0.32
**MP/SN~ (degrees)**	33.3 8.1	33 6.5	0.89
**occ/SN~ (degrees)**	15 4.8	15.2 4.4	0.85
WITS (mm)	4.2 1.5	3.9 1.7	0.53
WITS_CR (mm)	4.4 1.3	3.9 1.5	0.21
ANS-Gn (mm)	66.2 6.1	65.9 5.6	0.85
ANS-Gn_CR (mm)	64.6 6.3	65.1 5.6	0.75
ANS-Me (mm)	64.5 5.3	63.6 6	0.53
ANS-Me_CR (mm)	63.1 5.6	62.8 5.9	0.82
Co-Gn/Co-A (mm/mm)	25.7 4.7	25.9 5.3	0.7
Co-Gn/Co-A_CR (mm/mm)	24.5 4.5	25.5 5.3	0.92
overjet (mm)	4.6 1.9	3.6 1.2	0.0164*
overjet_CR (mm)	5.5 2	3.7 1.2	0.0001***
overbite (mm)	1.6 2.2	2.3 2.2	0.38
overbite_CR (mm)	3 2.1	3.2 2.2	0.78

~variables that remain constant during CO-CR conversion; SD - standard deviation; *$p < 0.05$; ***$p < 0.001$; CR - centric relation.

Table S3. Results of linear measurements of joint space in saggital plane.

Variable	study group mean SD (mm)	control group mean SD (mm)	p-value
R (SS)	2.5 0.9	3 0.7	0.031*
R (AS)	2 0.7	2.2 0.8	0.3
R (PS)	1.9 0.8	2.1 0.7	0.12
L (SS)	2.7 1.1	2.9 0.9	0.34
L (AS)	2.1 0.9	2.2 0.8	0.6
L (PS)	2 0.8	2.3 0.8	0.3

R- right joint; L- left joint; PS - posterior joint space; SS - superior joint space; AS - anterior joint space; *$p < 0.05$.

Table S4. Displacement of condylar processes in the maximal intercuspation position.

Variable	study group mean SD (mm)	control group mean SD (mm)	study group median [min, max]	control group median [min, max]	p-value
Δx R	-0.35 1.6	0.1 1.3	-0.25 [-3.5; 2.5]	0 [-2; 2.75]	0.33
Δx L	-0.47 1.7	-0.1 1.2	-0.63 [-3; 2.3]	-0.2 [-2.2; 2.5]	0.19
Δz R	1.7 1.1	1.2 1	1.63 [-0.3; 3.5]	1 [-0.2; 3.2]	0.06
Δz L	1.8 1.2	1.4 0.9	1.85 [-0.2; 3.5]	1.5 [-0.1; 3]	0.22
Δy	0.4 0.9	0.1 0.85	0.4 [-1.5; 2]	0.1 [-2; 2.25]	0.22

SD- standard deviation; R- right side; L- left side; Δx- condylar displacement in anteroposterior axis; Δz- condylar displacement in transverse axis; Δy- condylar displacement in vertical axis.

Table S5. Results of CO-CR conversion of cephalometric linear and angular measurements in study group.

Cephalometric variable	difference between means SD	p-value
SNB_CR vs SNB (degrees)	-0.43 0.94	0.65
ANB_CR vs ANB (degrees)	0.58 0.69	0.4
WITS_CR vs WITS (mm)	0.13 0.83	0.87
ANS-Gn_CR vs ANS-Gn (mm)	-1.6 1.61	0.32
ANS-Me_CR vs ANS-Me (mm)	-1.38 1.42	0.34
Co-Gn/Co-A_CR vs Co-Gn/Co-A (mm/mm)	-1.23 1.19	0.31
overjet_CR vs overjet (mm)	0.89 0.50	0.08
overbite_CR vs overbite (mm)	1.38 0.56	0.016*

SD- standard deviation; *$p < 0.05$; CR - centric relation.

Table S6. Results of CO-CR conversion of cephalometric linear and angular measurements in control group.

Cephalometric variable	difference between means SD	p-value
SNB_CR vs SNB (degrees)	-0.01 0.93	0.99
ANB_CR vs ANB (degrees)	0.12 0.47	0.81
WITS_CR vs WITS (mm)	-0.03 0.59	0.96
ANS-Gn_CR vs ANS-Gn (mm)	-0.85 1.45	0.56
ANS-Me_CR vs ANS-Me (mm)	-0.79 1.53	0.61
Co-Gn/Co-A_CR vs Co-Gn/Co-A (mm/mm)	-0.41 1.36	0.77
overjet_CR vs overjet (mm)	0.16 0.31	0.62
overbite_CR vs overbite (mm)	0.87 0.56	0.13

SD- standard deviation; CR - centric relation.

Table S7. Comparison of cephalometric linear and angular measurements in study group with CD>=2 according to Δx i Δz.

Cephalometric variable	p-value Δx L	p-value Δx R	p-value Δz L	p-value Δz R
SNB_CR	0.5	0.18	0.92	0.61
ANB_CR	0.0057*	0.048*	0.3	0.26
WITS_CR	0.08	0.31	0.64	0.34
ANS-Gn_CR	0.84	0.32	0.08	0.77
ANS-Me_CR	0.77	0.23	0.23	0.91
Co-Gn/Co-A_CR	0.24	0.78	0.88	0.78
overjet_CR	0.0002***	0.0002***	0.0013*	0.0068*
overbite_CR	0.39	0.88	0.77	0.97

L - left condyle; R - right condyle; Δx- condylar displacement in anteroposterior axis; Δz- condylar displacement in transverse axis; *p < 0.05; ***p < 0.001; CR - centric relation.

Table S8. Correlations of cephalometric linear and angular measurements as well as condylar displacement in study group with Δx i Δz.

Variable/type of condylar displacement	type of condylar displacement	p-value	r-value
ANB_CR	Δx L	0.008*	0.45
ANB_CR	Δx R	0.012*	0.46
ANS-Gn_CR	Δx R	0.015*	0.44
ANS-Me_CR	Δx R	0.005*	0.50
ANS-Me_CR	Δz L	0.049*	-0.36
Co-Gn/Co-A_CR	Δx L	0.019*	-0.43
Δx L	Δx R	0.004*	0.51
Δz L	Δz R	1.52E-06***	0.75

r-value - correlation coefficient; L - left condyle; R - right condyle; Δx- condylar displacement in anteroposterior axis; Δz- condylar displacement in transverse axis; *p < 0.05; ***p < 0.001; CR - centric relation.

Table S9. Correlations of cephalometric linear and angular measurements as well as condylar displacement in control group with Δx i Δz.

Variable/type of condylar displacement	type of condylar displacement	p-value	r-value
SNA	Δz R	0.024*	0.41
SNB_CR	Δx L	0.043*	-0.37
MP/SN	Δx R	0.019*	-0.43
overbite_CR	Δz R	0.015*	0.49
Δx L	Δx R	0.014	0.45
Δx L	Δz L	0.008*	-0.47
Δx L	Δz R	0.004*	-0.51
Δz L	Δz R	4.96E-05***	0.67

r-value - correlation coefficient; L - left condyle; R - right condyle; Δx- condylar displacement in anteroposterior axis; Δz- condylar displacement in transverse axis; *p < 0.05; ***p < 0.001; CR - centric relation.

Table S10. Correlations of cephalometric linear and angular measurements as well as condylar displacement and joint space in study group.

Variable/joint space	joint space	p-value	r-value
SNB_CR	L(SS)	0.046*	0.37
PP-MP	L(SS)	0.027*	-0.41
SN/GoGn	R(SS)	0.027*	-0.40
SN/GoGn	L(SS)	0.012*	-0.45
PP/GoGn	R(SS)	0.003*	-0.52
PP/GoGn	L(SS)	0.01*	-0.46
FMA	R(SS)	0.002*	-0.55
FMA	L(SS)	0.001*	-0.58
MP/SN	R(SS)	0.016*	-0.43
MP/SN	L(SS)	0.011*	-0.46
overjet_CR	L(SS)	0.014*	-0.44
overjet_CR	L(AS)	0.05*	-0.36
overbite_CR	R(PS)	0.017*	-0.43
overbite_CR	L(PS)	0.017*	-0.43
R(SS)	R(PS)	0.012*	-0.45
R(SS)	L(SS)	0.038*	0.38
R(AS)	L(AS)	3.39E-05***	0.68
R(PS)	L(SS)	0.011*	0.46
R(PS)	L(PS)	0.002*	0.54
L(SS)	L(PS)	6.63E-10***	0.87
Δz	R(PS)	2.81E-04***	0.62
		0.025*	0.41

r-value - correlation coefficient; L - left condyle; R - right condyle; *p < 0.05; ***p < 0.001; CR - centric relation; R- right joint; L- left joint; PS - posterior joint space; SS - superior joint space; AS - anterior joint space; Δz- condylar displacement in transverse axis.
